# Coexisting Benign Tumors in a Finger Are Rare but Not Impossible

**DOI:** 10.7759/cureus.37863

**Published:** 2023-04-20

**Authors:** Efstratios D Athanaselis, Efstathios Konstantinou, Alexandros Koskiniotis, Theofilos Karachalios, Sokratis Varitimidis

**Affiliations:** 1 Department of Orthopaedic Surgery and Musculoskeletal Trauma, University Hospital of Larissa, Larissa, GRC

**Keywords:** benign hand tumors, bone grafts, excisional biopsy, enchondroma, giant cell tumor

## Abstract

Giant cell tumors of the tendon sheath (GCTTS) and enchondroma are identified as the most prevalent benign soft tissue and bone tumors of the hand. While their individual presence is a common finding, their concurrent appearance in the same anatomic region is exceptionally rare, making simultaneous diagnosis more burdensome. We present a noteworthy case of GCTTS and enchondroma in the index finger of a young patient, along with the therapeutic strategy for correct diagnosis and effective treatment of such an occurrence.

## Introduction

Giant cell tumors of the tendon sheath (GTTS) and enchondromas are identified as the most prevalent benign soft tissue and bone tumors of the hand, respectively, with the fingers being the most frequent location [1, 2].

A giant cell tumor of the tendon sheath in the hand is a benign tumor that invades the soft tissues as well as the adjacent joints. The radial three fingers, especially the index finger, are most commonly affected, and there is a considerable risk of recurrence [3, 4]. According to the World Health Organization, GCTTS is categorized in the group of fibrohistiocytic tumors, which are considered to be of low malignant potential. Though in most cases, the very concept of fibrohistiocytic differentiation is somewhat ambiguous, the histiocytic component is non-neoplastic but substantially contributes to the development of the lesion, as in giant cell tumors (GCT) [5].

Solitary enchondroma is a benign bone tumor, often found incidentally on radiographs, computed tomography, or magnetic resonance imaging [6]. It is composed of lobules of hyaline cartilage, consisting of benign chondrocytes, and can be surrounded by reactive bone formation [7]. Enchondromas are typically asymptomatic, except in cases of extensive lesions, which may be painful, cause deformity, or result in pathologic fractures [6].

While GCTTS and enchondromas are common benign tumors of the hand, their coexistence in close proximity at the same location is remarkably rare. We present such an unusual case of GCT and enchondroma in the index finger, treated in our orthopedic department.

## Case presentation

A 23-year-old woman, right-hand dominant, presented to our hospital with a two-month history of pain over the proximal interphalangeal joint and the proximal phalanx of the left index finger. She also referred to a palpable, painless mass at the pulp of the same finger that had been growing for the last two years. She mentioned that, although the mass at the pulp of the index had concerned her, she had not consulted a doctor because of the COVID-19 pandemic.

Physical examination revealed a palpable lump with a diameter of 10-15 mm on the volar side of the tip of the distal phalanx and circumferential swelling of the proximal phalanx of the index finger. Palpation of the proximal phalanx and proximal interphalangeal (PIP) joint of the index was painful, and the PIP range of motion was limited between 10^o^ to 80^o^. The patient's medical history was free of any injury, disease, or previous surgery.

**Figure 1:**
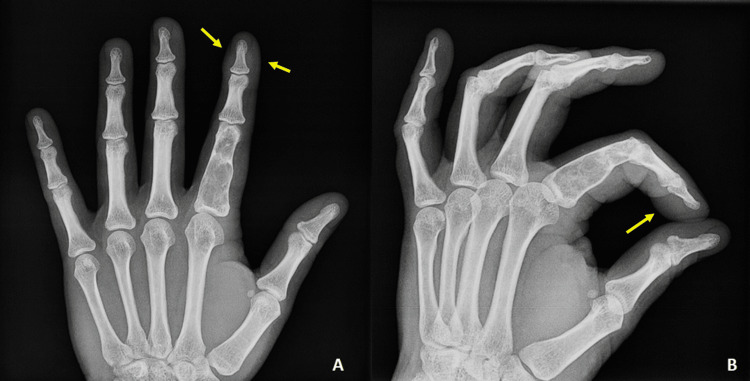
Face (A) and profile (B) x-rays of the left index finger. Enchondroma is obvious in both X-rays. The distal phalanx lump is nearly marked in both X-rays (arrows).

Radiographs of the index finger revealed a well-defined radiolucent lesion occupying the whole proximal phalangeal bone with internal septations by thinned cortex (Figure 1). No periosteal reaction, calcification of the tumor matrix, or joint involvement was marked.

**Figure 2:**
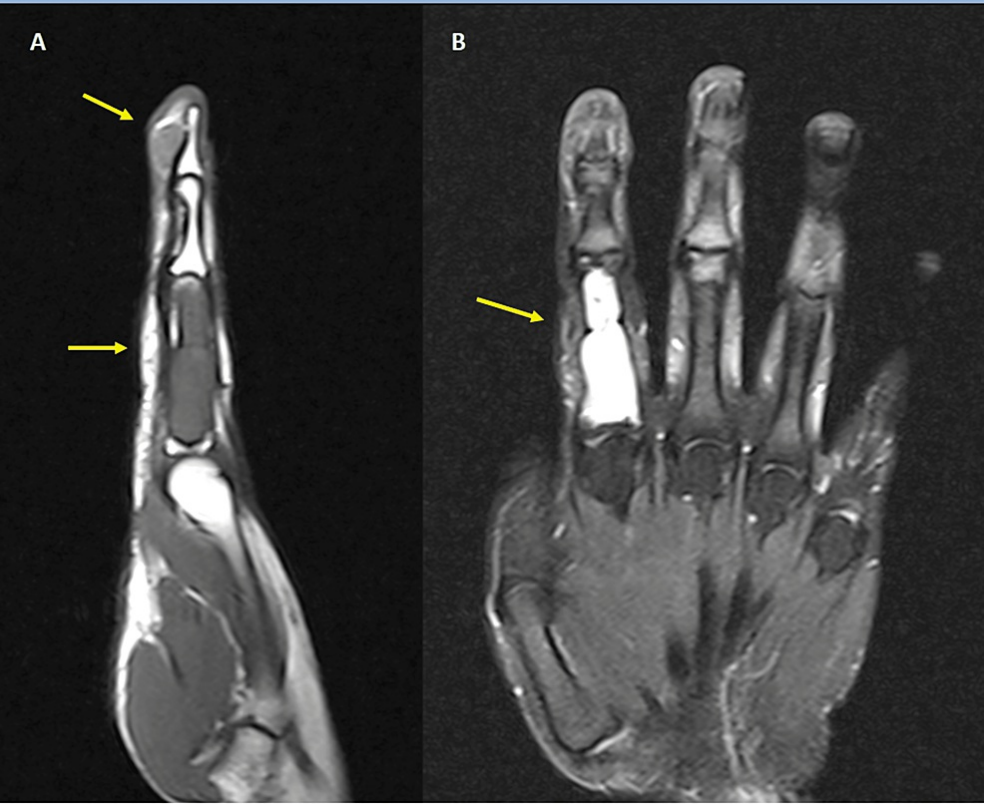
T1-weighted (A) and T2-weighted (B) MRI images of the index finger

A soft tissue mass was scarcely identifiable on the volar aspect of the distal phalanx in X-rays. An MRI performed to further investigate these lesions on the index finger confirmed the involvement of the whole proximal phalanx without any extension to the surrounding tissues (Figure 2).

**Figure 3:**
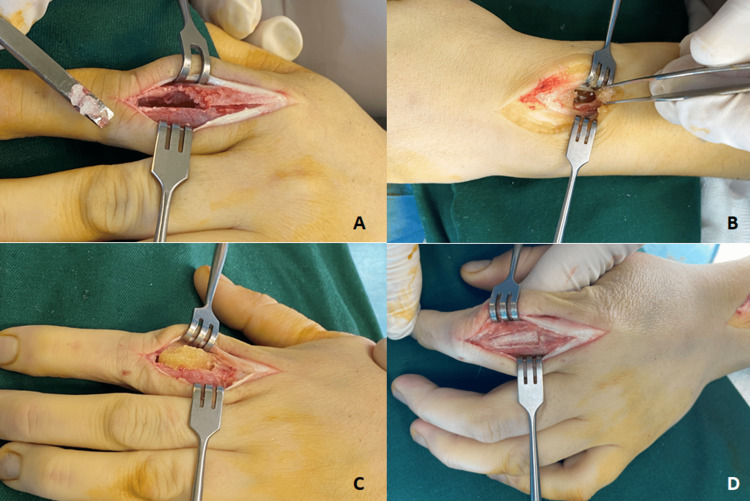
(A) Dorsal approach, fenestration of the proximal phalanx, and extraction of cartilaginous pathological tissue. (B) distal radius donor site for autografts. (C) The medullary cavity is filled with autografts and DBM paste. (D) The cortical window is closed.

Excisional biopsy of the soft tissue mass and curettage of the bone lesion of the proximal phalanx were performed, followed by autografting using cancellous bone harvested from the ipsilateral radius by creating a window on the dorsal cortex of the distal metaphysis just proximal to the Lister tubercle. The demineralized bone matrix (DBM) paste was mixed with the cancellous bone (Figure 3).

**Figure 4:**
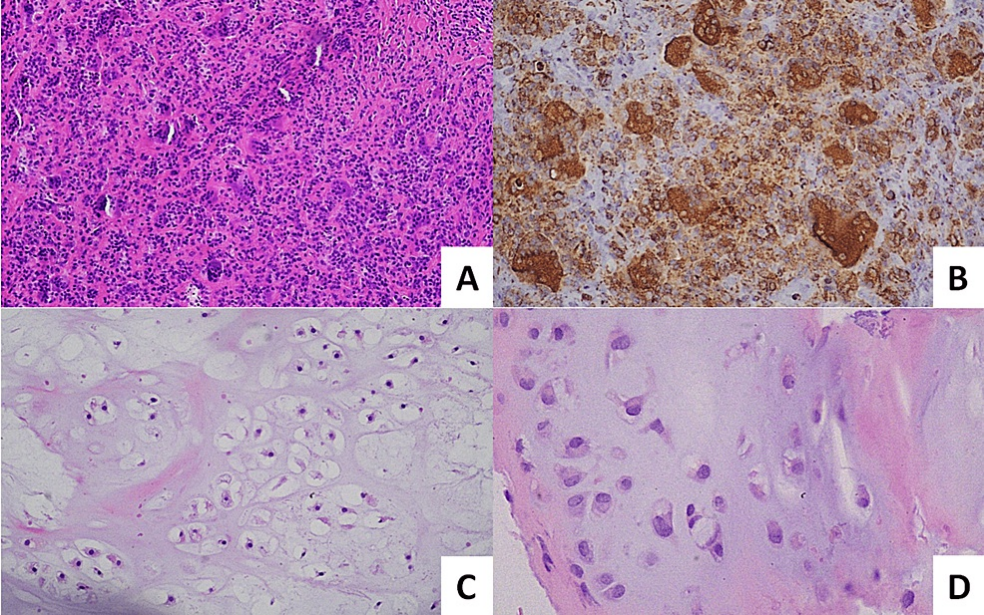
Microscopic examination of the excised lesions revealed the following regarding GCT: (A) an admixture of giant cells, foamy macrophages, and round mononuclear cells, along with areas with cleft-like spaces (hematoxylin and eosin stain, 100x magnification); (B) giant cells and foamy macrophages stained positively with immunostain for CD68 (200x magnification). Regarding the enchondroma, there is a mass of hyaline cartilage in lobular formation, areas with myxoid change of the stroma, abundant hyaline cartilaginous matrix (hematoxylin and eosin stain, 200x magnification) (C), and chondrocytes with densely staining nuclei and small eosinophilic cytoplasm arranged in the lacunae in a haphazard fashion (hematoxylin and eosin stain, 400x magnification) (D).

The first gross specimen, the soft tissue mass, was a well-circumscribed, yellowish-brown piece of tissue measuring 16x8x7mm. Microscopically, the tissue was totally compatible with GCTTS. The second specimen from the intraosseous lesion of the proximal phalanx was a white cartilaginous material measuring roughly 2-3cm^3^. Microscopically, the tumor was composed of mature cartilage lobules, with the presence of double-nuclei chondrocytes showing no mitotic activity (Figure 4). The histopathological report concluded that no malignant features were observed in both specimens and certified the enchondroma and GCTTS diagnoses.

**Figure 5:**
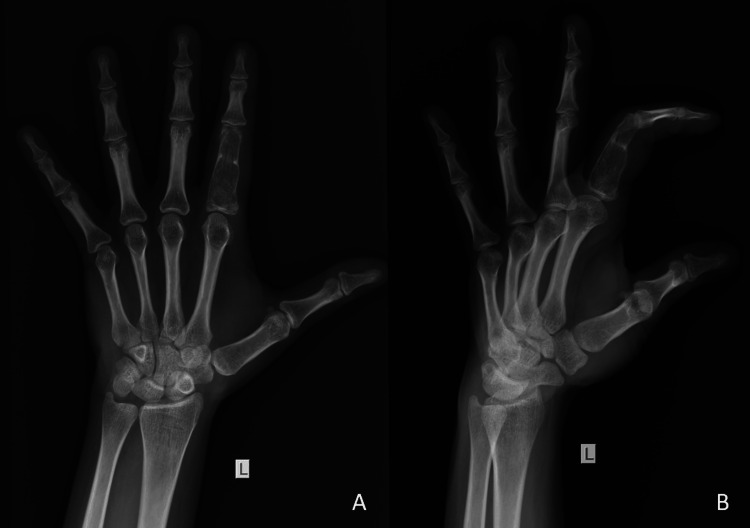
Face (A) and profile (B) x-rays of the hand, five weeks postoperatively

During the most recent follow-up visit one year postoperatively, surgical wounds were completely healed, grafts had been consolidated, and the normal range of motion of the PIP joint had been regained. The patient had returned to activities of daily living with satisfactory, painless function. No evidence of recurrence was observed by clinical and imaging assessments (Figure 5).

## Discussion

GCTTS accounts for 74.2% of all benign soft tissue tumors in the hand [8], with a predominant distribution in the distal joint of the finger [9]. Plain radiographs typically demonstrate a soft tissue mass and bone changes, including osseous invasion, periosteal reaction, cystic changes, and calcification. CT and MRI scans usually show a solid, non-homogeneous mass, which can also present hemorrhagic features in rare cases [10, 11]. Phalangeal bone involvements of GCTTS may mimic a primary bone tumor, appearing as an intraosseous lytic lesion on radiographs; however, this is rare [12]. The presence of necrosis, metaplastic bone growth, and aneurysmal bone cyst-like lesions can be used to microscopically differentiate giant cell tumors of bone from GCTTS. However, fibromas of the tendon sheath and extra-skeletal osteosarcomas should always be considered [13, 14].

Enchondroma is the most frequent benign bone tumor of the hand, constituting 35%-65% of cases. The most common location is the little finger, as interpreted in the meta-analysis of 327 cases by Gaulke et al. [2]. A solitary enchondroma may progress very slowly and remain asymptomatic until the phalanx is significantly expanded and deformed. The thinning of the bone cortex causes pain due to an imminent fracture or instability. Deformity of the bones and pain are the main symptoms that make a patient seek medical consultation. The typical appearance of an enchondroma on an X-ray is that of a well-defined lytic tumor, with thinning or disruption of the cortices in more advanced cases, in which osteosarcoma should always be included in the differential diagnosis. An MRI scan can provide valuable information regarding the relationship of the tumor with the surrounding soft tissue and help us narrow the differential diagnosis by depicting some particular tumor characteristics, like fluid levels in the case of a cyst [15]. The risk for malignant transformation of an enchondroma into a chondrosarcoma is exceptionally low in the hand. However, recurrence can appear after operative treatment with a rate ranging from 2% to 15% in the literature [16].

The surgical treatment of GCTTS consists of dissection and excision of the entire lesion, taking care to protect and preserve the surrounding soft tissue, especially arteries, and nerves. The tumor is often strictly attached to the tendon sheath, pulleys, or volar plate, thus the excisional margins are not well-defined. The remaining cells can be the reason for GCTTS reappearance. As for the recurrence rate of these tumors, various factors have also been described, including pressure erosions on X-rays, lesions located at the interphalangeal joint, and the presence of degenerative osteoarthritis. Lowyck et al. did not manage to find a significant association between recurrence rate and these factors, and it is considered by the majority of the authors that only complete excision can achieve the best results regarding the reappearance of the tumor [17].

S. S. Suresh and Hosam Zaki published in 2010 a case series of 12 patients who underwent excision of GCTTS with only one case of recurrence in a follow-up period of three to nine years. The authors recommend that the use of an operating microscope or magnifying loupe could ensure radical excision of the main lesion and any possible satellite nodules [18].

On the other hand, enchondroma excision is carried out by fenestration of the bone cortex, meticulous extraction of the pathologic cartilaginous tissue, washout of the cavity with ethanol, and filling it with grafts. Consolidation of the grafts will enhance bone strength, avoiding pathological fractures, although there are authors suggesting that additional bone grafting may be unnecessary and should be used in specific indications [19]. In the case of hand enchondroma, autologous bone grafts can be harvested from the distal radius or the iliac crest. However, bone graft substitutes have shown comparable functional and radiological results [20] while eliminating the donor site morbidity of autologous grafting procedures. The use of both grafts in combination allows the filling of larger cavities and defects, utilizing their advantages. On the other hand, cases of spontaneous regression of the tumor during bone healing after a fracture have been reported.

The presented case shows that the coexistence of more than one different tumor in the hand, though very rare, cannot be ruled out [15]. The diagnosis of more than one coexisting tumor permits simultaneous operative treatment, decreasing cost and patient inconvenience. Both procedures can take place in a day-case surgery mode under local or regional anesthesia.

However, multiple lesions in a specific body region can suggest malignancy (skip lesions), and this must always be considered in the differential diagnosis of tumors, even in the hand where, in the vast majority of cases, such lesions are benign. Therefore, though malignancy can be excluded by a thorough preoperative MRI examination and intraoperative macroscopic findings in most cases, lesions must be excised with fine surgical technique and always be sent for pathoanatomical investigation.

## Conclusions

Benign tumors of the hand are not uncommon, and giant cell tumors of the tendon sheath and enchondroma are among the most common. The coexistence of these two different tumors in the same anatomic region as a finger, though seldom reported, must not elude doctors. A thorough investigation of the hand by physical and imaging examination can reveal additional, unnoticed lesions apart from the patient's main complaint. This can allow simultaneous treatment of tumors in a single operation.
